# Membranes in Water Reclamation: Treatment, Reuse and Concentrate Management

**DOI:** 10.3390/membranes13060605

**Published:** 2023-06-15

**Authors:** Sukanyah Devaisy, Jaya Kandasamy, Tien Vinh Nguyen, Harsha Ratnaweera, Saravanamuthu Vigneswaran

**Affiliations:** 1 Faculty of Engineering, University of Technology Sydney (UTS), P.O. Box 123, Broadway, NSW 2127, Australia or sukanyahdev@vau.ac.lk (S.D.); jaya.kandasamy@uts.edu.au (J.K.);; 2 Department of Bio-Science, Faculty of Applied Science, University of Vavuniya, Vavuniya 43 000, Sri Lanka; 3 Faculty of Sciences & Technology (RealTek), Norwegian University of Life Sciences, P.O. Box 5003, NO-1432 Ås, Norway

**Keywords:** membrane, reverse osmosis, hybrid membrane systems, concentrate, wastewater, water reclamation

## Abstract

In this article, an extensive examination is provided on the possible uses of membranes and hybrid processes in wastewater treatment. While membrane technologies face certain constraints, such as membrane fouling and scaling, the incomplete elimination of emerging contaminants, elevated expenses, energy usage, and brine disposal, there are approaches that can address these challenges. Methods such as pretreating the feed water, utilizing hybrid membrane systems and hybrid dual-membrane systems, and employing other innovative membrane-based treatment techniques can enhance the efficacy of membrane processes and advance sustainability.

## 1. Introduction

Ensuring global sustainability relies heavily on the availability of safe water [1]. Consequently, it becomes imperative to safeguard secure water sources. An attractive solution for extending current water supplies is the reuse of treated municipal wastewaters for nonpotable purposes. Nowadays, properly treated wastewater is considered an alternative water source. Shannon et al. [2] have recognized water reuse as a scientific pursuit, with the reclamation of biologically treated sewage effluent (BTSE) being a significant strategy for water security; however, the complex organic composition of BTSE can hinder the efficacy of water treatment, resulting in subpar quality for water reuse [3,4]. To tackle this challenge, a future direction for water reclamation and reuse involves capturing water directly from industrial or municipal wastewaters and reclaiming it to meet drinking water standards. Apart from BTSE, wastewaters produced from industrial processes also undergo membrane-based treatments to enable their reuse purposes.

The utilization of membrane technology provides a significant benefit in the realm of consistent water quality. Membrane processes excel at effectively separating contaminants and pathogens, making them well suited for water reuse scenarios that demand high-quality water. Moreover, membrane filtration requires less physical space and labor, can be readily automated, and surpasses conventional filtration methods in terms of contaminant removal.

In contrast to other wastewater treatment technologies, membrane filtration is less expensive. This is because of its lower installation costs, lower energy costs, and fewer processing steps. At the same time, it produces high-quality water suitable for reuse. The worldwide membrane bioreactor (MBR) market size in wastewater reclamation rose from USD 0.25 to 1.17 billion from 2006 to 2010 [5]. Initially, the energy requirements for the air scouring of MBRs have been a limiting factor. Thanks to research, air scouring for submerged hollow fiber MBRs represents only 20% of the energy required [5]. With significant reductions in the size of equipment, lower energy requirements, and low capital costs, membrane technology becomes an attractive solution in wastewater treatment [6,7,8].

On the other hand, nanofiltration (NF) is commonly used to address water hardness due to its lower labor and operation costs in comparison to chemical methods [9,10]. NF membranes with a pore size of approximately 1 nm can effectively remove relatively smaller organic substances such as pharmaceuticals and personal care products (PPCPs), color, degradation products from BTSE, and dissolved organics, targeting dissolved compounds [11] with a molecular weight of about 300 Da. NF proves to be an efficient system capable of treating BTSE to produce water of desired quality for industrial, agricultural, and indirect potable reuse applications [8,10].

Reverse osmosis (RO) has been widely adopted in water reclamation and reuse since the 1970s [12]. It excels at removing salts and low-molecular-weight compounds from wastewaters [13], producing high-quality water suitable for reuse purposes. RO membranes, which are dense and without predefined pores, exhibit slower permeation. Rejection in RO is not based on sieving but rather on a solution–diffusion mechanism. The low permeability of RO membranes necessitates high pressure, typically ranging from 20 to 120 bar [14].

However, membrane fouling poses a primary challenge in membrane technology, leading to reduced filtration performance [15]. It affects filtration flux, the contaminant removal rate, and diminishes the lifespan of membranes, ultimately increasing costs. Therefore, periodic chemical cleaning is crucial for sustainable operation [15]. Alongside membrane fouling, effectively managing and disposing of RO concentrates (ROCs) and the substantial energy consumption required are other significant challenges associated with membrane technology [16]. ROCs contain all of the contaminants, including organics, nutrients, and emerging contaminants, that are rejected by RO. If not properly disposed of, ROCs can pose risks to human and ecological health [17,18].

Modifying the surface properties of membranes is essential for reducing fouling and has a significant impact on selectivity and flux [15]. Several studies have been conducted to reduce fouling through membrane surface modification [19,20,21,22,23,24]; however, achieving improved membrane performance solely through a single membrane treatment technology poses challenges. It is more feasible to optimize different types of membranes using physicochemical treatments to enhance their performance. Therefore, there is a need for research focusing on innovative membrane processes, appropriate pretreatment methods, and membrane optimization for water reclamation and reuse, both from BTSE and other industrial processes. Previous reviews on membrane-based treatments for wastewater reclamation, high-quality water reuse, and ROC management have been limited in scope.

Therefore, this review provides a critical evaluation and comprehensive overview of the following aspects: (1) membrane-based water treatment strategies that integrate physicochemical treatment techniques to enhance membrane performance, (2) high-quality water reuse achieved through hybrid dual-membrane systems and innovative membrane treatment approaches, (3) membrane-based treatments for effectively managing membrane-rejected concentrates, such as ROCs, and (4) advanced membrane bioreactor hybrid systems. Given the limited scope of previous review articles on these topics, this review aims to assist researchers in identifying gaps in membrane treatment and informing their future work.

## 2. Membranes for Water Reclamation

This section reviews the integration of physicochemical treatments and biological treatments with membrane technologies as a pretreatment and hybrid form to treat wastewater for the removal of contaminants for reuse purposes.

### 2.1. Membranes with Physicochemical Treatments

The application of conventional/physicochemical treatment technologies, such as adsorption, coagulation, preoxidation, ion exchange, membrane filtration, and softening, are applied as pretreatments before membrane filtration [25]. This reduces the organic loading of feed water and minimizes the effects of fouling and scaling, while enhancing contaminant removal efficiency. Pretreatments also contribute to energy utilization [7].

Coupling MF/UF membranes with these treatments creates membrane hybrid systems [26], which simplify the membrane process, reduce fouling effects, and extend membrane lifespan and replacements.

As reported by Loganathan et al. [27], there are different configurations of membrane systems:

- Pretreatment: Treatment prior to membrane filtration.
- Hybrid systems: Treatment with membrane filtrations.
- Post-treatment: Treatment after membrane filtration.

**Figure 1:**
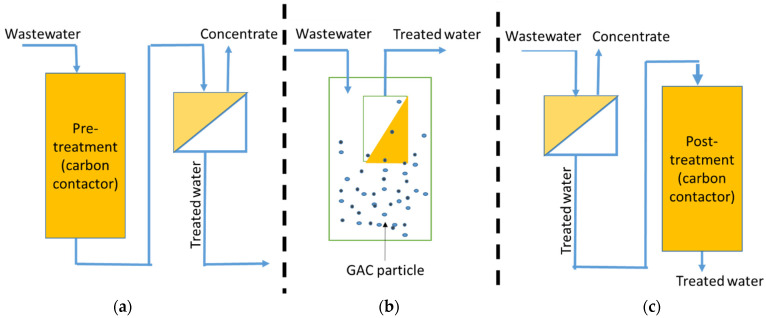
Different configurations of hybrid membrane systems: (**a**) pretreatment, (**b**) hybrid, and (**c**) post-treatment (modified and redrawn from Loganathan et al. [27]).

Figure 1a shows a pretreatment where the wastewater is firstly passed through to remove contaminants prior to membrane filtration. This enables the system to optimize removal efficiency by changing the physicochemical characteristics of the treatment system prior to the membrane.

Figure 1b illustrates a hybrid system where physicochemical treatments (such as adsorbents/ion exchangers or other chemicals) are incorporated into a membrane reactor that contains the feed water requiring treatment. This process takes place in a single tank, resulting in a straightforward, one-stage process that offers superior physical removal of contaminants from wastewaters.

Figure 1c shows the third configuration; the post-treatment method involves passing the feed water through the membrane first, followed by the physicochemical treatment. However, a potential drawback of this method is that the treated water may contain residues of the adsorbents and chemicals used in the treatment process. To address this issue, a finely tuned physical separation barrier is required to prevent the export of fine carbon particles [27]. Since this configuration is not practical, it is not discussed in this review paper.

#### 2.1.1. Pretreatment to Membrane

**Figure 2:**
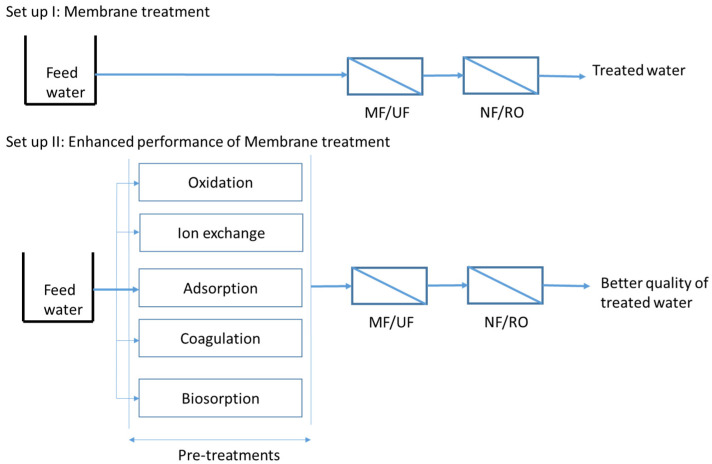
Different pretreatments applied prior to the membrane to enhance the performance of membrane filtration.

A pretreatment refers to any form of treatment administered prior to membrane filtration to eliminate contaminants that could potentially cause fouling or harm to the membrane. Various types of pretreatments, including oxidation, adsorption, ion exchange, coagulation, and biosorption, can be employed to mitigate membrane fouling and optimize energy utilization [28,29,30,31]. The implementation of such pretreatment methods improves the efficiency of membrane filtration and results in the production of higher quality treated water [32] (Figure 2).

Several physicochemical treatments applied before membrane filtration have been explored in previous research and described below. 

One such treatment is coagulation, which was investigated by Katalo et al. [33]. Their study focused on the use of *Moringa oleifera* and alum as coagulants to treat river water and remove foulants that could potentially lead to fouling effects on MF membranes. The findings revealed that the application of these coagulants before membrane filtration significantly reduced fouling effects.

Fan et al. [34] conducted a study on the impact of coagulation on the performance of MF and UF membranes in treating secondary effluent for reuse. The addition of alum at a dosage of 5 mg L^−1^ Al^3+^ resulted in significant flux improvement by mitigating membrane fouling. The highest removal of effluent organic matter (7.4%) was achieved at this alum dosage. An analysis of the organic composition revealed that alum effectively removed hydrophilic substances, particularly high-molecular-weight organic components (40–70 kDa), which are the main contributors to membrane fouling. Additionally, the study found that the removal of UV-absorbing organics was 8.2% for MF alone, while MF followed by coagulation at the mentioned dose resulted in a removal of 21.5%.

Elma et al. [35] demonstrated the application of coagulation as a pretreatment to enhance the performance of silica–pectin membranes for desalination. A coagulant dose of 30 g L^−1^ exhibited improved water flux at 60 °C, reaching 12.2 kg m^−2^ h^−1^.

Bouchareb et al. [36] reported the efficient removal of turbidity, COD, and color by using alum as a pretreatment to an NF90 membrane at 20 bar, achieving a removal efficiency of over 99%.

Ion exchange: A study investigated the effectiveness of a combined ion-exchange and NF membrane system for removing parabens from river water samples. The pretreatment involved using ion-exchange resins, specifically MIEX^®^ DOC and MIEX^®^ GOLD, before subjecting the water to two different NF membranes, namely NF-90 and DESAL-HL. The results demonstrated that the combined treatment utilizing both the ion-exchange resins and NF membranes achieved the complete removal (100%) of parabens. In contrast, when the NF membranes were employed alone, the removal efficiency of parabens was found to be 91–92% [37].

In the study by Fan et al. [34], it was noted that pretreatment with anion-exchange resins before MF/UF membrane filtration did not have a significant impact on flux improvement; however, it resulted in the highest removal (>50%) of organics from secondary effluent. The anion-exchange resin primarily removed lower molecular weight and negatively charged organic fractions, which are known to contribute to membrane fouling and subsequent declines in flux.

Yu et al. [38] investigated a continuous flow bench-scale UF membrane apparatus using synthetic water. They observed that a pretreatment combining MIEX and microsand with an alum pretreatment reduced UF membrane fouling by approximately 50% compared to a pretreatment with alum alone during 60 days of operation.

Zhang et al. [39] investigated the impact of incorporating a fluidized bed MIEX^®^ reactor before membrane filtration. To achieve this, they utilized synthetic wastewater with a DOC concentration of 10 mg/L as the feed water. The pretreatment process involved a fluidized bed MIEX^®^ reactor, with a volume of 100 mL and a fluidization depth of 80.3 cm, prior to the submerged membrane hybrid system.

Adsorption: Adsorption is recognized as one of the most cost-effective, efficient, and simple processes for removing organic micro pollutants (OMPs), and is widely utilized. It offers the advantages of minimal chemical or biological sludge waste generation, the absence of undesirable byproducts, and the ability to regenerate and reuse the adsorbent, thereby reducing operational costs [40]. Among various adsorbents, carbon-based materials, such as activated carbon, carbon nanotubes, graphene and its derivatives, biochar, engineered hierarchical porous carbon materials, and ion-exchange resins, have proven to be highly effective in removing OMPs [41,42].

Zhang et al. [42] found that a hybrid electrochemical microfiltration GAC adsorption (e-MF-GAC) pretreatment exhibited reduced fouling in reverse osmosis (RO) systems. The e-MF-GAC pretreatment demonstrated a 30% higher permeate flux compared to ultrafiltration (UF). Furthermore, the pretreatment effectively reduced the deposition of organic foulants on the membrane compared to UF.

The use of GAC as a pretreatment to a granular sludge sequencing batch reactor (GS-SBR)/MF system demonstrated improved performance of the MF membrane with reduced membrane fouling. Additionally, significant removals of organics and nutrients were observed, ranging from 27.3% to 32.1% [43]. In another study involving synthetic wastewater containing syntan with an initial concentration of 500 mg/L, a GAC column packed with 58 g of GAC achieved the removal of 55–70% of dissolved organic carbon (DOC) at a low filtration velocity of 0.5 m/h [44].

A summary of pretreatments applied prior to membranes are summarized in Table 1.

#### 2.1.2. Membrane Hybrid Systems (MHSs)

**Figure 3:**
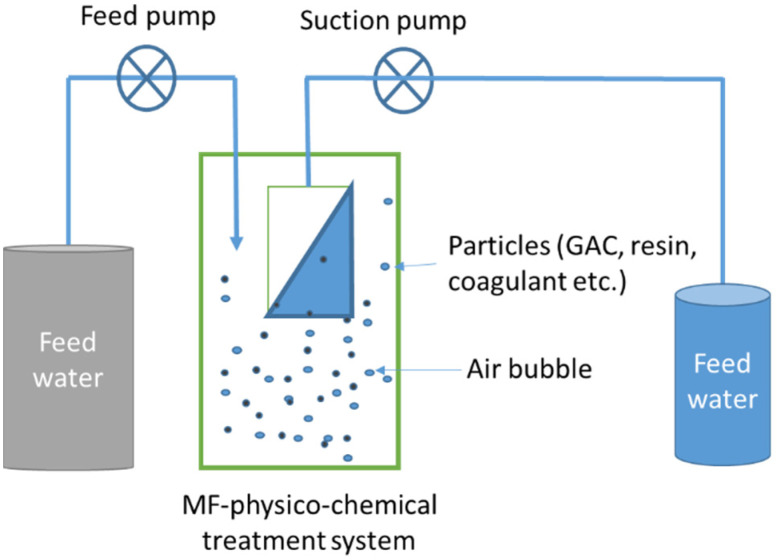
Hybrid membrane systems integrated with physicochemical treatment methods (modified and redrawn from Devaisy et al. [45]).

Figure 3 illustrates the integrated membrane–hybrid system, which combines the membrane process with physicochemical methods such as adsorption, ion exchange, coagulation, bioconversion, and catalysis [27,45,46].

By using hybrid membrane systems, membrane fouling is minimized, which reduces operation costs by reducing the frequency of membrane cleaning and extending membrane life; however, the efficiency of physicochemical treatments in removing contaminants and minimizing membrane fouling is influenced by several factors, such as the type of agent, dosage, dosing modes (continuous or intermittent), properties of wastewaters (colloidal/dissolved/bulk/synthetic organics/inorganics), and solution chemistry [47].

Aeration and the particle size of adsorbents also play a critical role in reducing membrane fouling through abrasion and scouring [45,48,49].

Impact of aeration: To minimize fouling on the membrane surface, aeration is provided into a membrane reactor to optimize the adsorption of potential foulants from feedwater and keep the carbon particles in suspension [32]. The air bubbles generated by aeration also reduce the deposition of solid particles onto the membrane surface through air scouring effects [50,51]. Pradhan et al. [52] found that doubling the air flow rate from 600 to 1200 L/h/m^2^ reduced TMP development by 32% at a filtration flux of 10 L/m^2^.h. Similarly, Johir et al. [53] reported that increasing the aeration rate from 1 to 1.5 m^3^/(m^2^ membrane area h) reduced TMP at a flux of 25 L/m^2^ h.

Particle sizes of adsorbents: Studies have also shown that the particle size of adsorbents can influence membrane fouling and TMP development [54,55]. For instance, Johir et al. [54] found that GAC particles with a size of 300–600 µm were more effective in reducing membrane fouling compared to particles with sizes of 150–300 µm or 600–1200 µm.

##### Membrane–Adsorption (GAC) Hybrid System

Previous studies have explored the utilization of activated carbon in hybrid membrane systems to mitigate membrane fouling and transmembrane pressure (TMP) development [55,56]. As reported by Aslam and Kim [55], the TMP value in an anaerobic fluidized bed bioreactor (AFBR) reached approximately 0.3 bar within a day of membrane operation when GAC fluidization was not applied; however, with GAC fluidization, membrane fouling was significantly reduced at a permeate flux of 15 L/m^2^ h. The membrane used had a pore size of 0.1 μm.

Hilbrandt et al. [57] investigated the impact of iron hydroxide adsorbents on the removal of phosphates in a submerged ultrafiltration (UF) membrane reactor. The hybrid system demonstrated no fouling issues, even with the use of up to 6.3 g/L adsorbent. 

For instance, Vigneswaran et al. [58] found that a 5 g/L dose of powdered activated carbon (PAC) in a membrane–PAC hybrid system reduced TOC removal by 84% over 15 days. A bench-scale hybrid system coupled with PAC showed no removal of organics in the absence of PAC, and a higher dose of PAC (40 g/L) only achieved 85% removal of dissolved organics [59]. PAC was found to remove low-molecular-weight organics, such as humics, that are smaller than the pore size of MF membranes, and the PAC particles can be fully retained by the MF [60].

Additionally, Johir et al. [54] observed that a GAC particle size of 300–600 µm was more effective in minimizing TMP development (16 kPa). Another study on an MBR with GAC in suspension showed a 50% decrease in TMP development [53].

##### Membrane–Ion-Exchange (PuroliteA502PS) Hybrid System

The integration of IEXs has not been extensively studied. Humbert et al. [61] studied the impact of various particle sizes of IER (<50; 50–100; 100–200; and 200–400 μm), where AER’s smaller particle size exhibited a better removal of organics than larger particles with a short contact time of less than 15 min. When combined with a hybrid membrane system, the removal of organics was significantly higher than that of the membrane alone; however, the use of smaller IEX particles (<50 µm) resulted in a relatively severe decline in flux (>60%) compared to larger particle sizes. A similar phenomenon was observed by our previous studies, where the effect of an MF–ion-exchange hybrid system for different sizes of IER (Purolite A502PS) in terms of DOC removal was studied [45,62]. A smaller particle size (150–300 µm) achieved better removal of DOC, whilst a larger particle size (425–600 µm) achieved lower removal of DOC. On the contrary, the TMP development with larger particles was less than that of smaller particles. A likely cause for this is the blocking of membrane pores by small Purolite A502PS particles, which inevitably reduces the membrane’s flux. 

Electrostatic interactions between charged trace organics and polymer resins with opposite charges have been found to exhibit stronger adsorption compared to activated carbon. Polymer resins have demonstrated the effective removal of charged heavy metals from water [63,64] and emerging organic contaminants [37,65,66] through electrostatic interactions; however, limited data are available regarding the removal of pharmaceuticals by ion-exchange polymer resins through such electrostatic interactions.

### 2.2. Membranes with Biological Treatment

Advanced membrane bioreactor hybrid systems that integrate membrane filtration with a biological treatment have emerged as viable options for water reclamation [67]. This approach generates an effluent that is nearly devoid of suspended solids, microorganisms, and organic micropollutants (OMPs), while offering a smaller footprint and lower costs for sludge disposal compared to conventional biological treatment methods.

**Figure 4:**
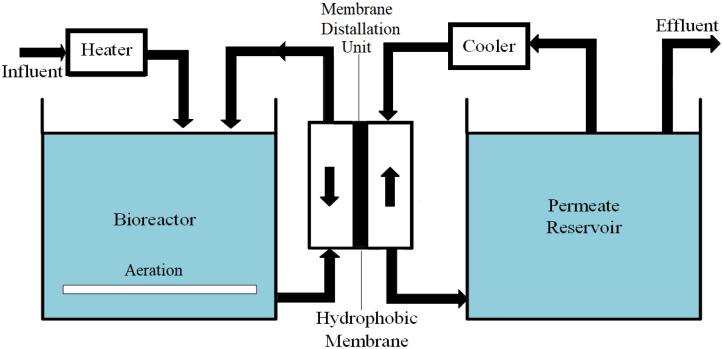
Schematic diagram of a sidestream MDBR process (modified and redrawn from Pathak et al. and Neoh et al. [68,69]).

By employing hybrid MBR systems, the quality of the treated effluent can be further improved while reducing membrane fouling and the frequency of cleaning operations [68]. Two notable advanced hybrid MBR systems are osmotic membrane bioreactors (OMBR) and membrane distillation bioreactors (MDBRs), both of which have demonstrated effectiveness in wastewater treatment. For a comprehensive understanding and comparison of these systems, Pathak et al. [69] provide a detailed description in their study (Figure 4).

#### 2.2.1. Osmotic Membrane Bioreactor

To reclaim and reuse indirect and direct potable water sources, osmotic membrane bioreactors (OMBRs) are utilized in wastewater treatment systems. These systems combine a bioreactor with semipermeable forward osmosis membranes. OMBRs are capable of producing better permeate quality with lower dissolved organic matter, a lower fouling tendency, and a higher reversibility of membrane fouling, as well as the improved removal of organic micropollutants [67]. Pathak et al. [69] provide recently published OMBR studies on the removal of organic micropollutants.

#### 2.2.2. Membrane Distillation Bioreactor

A hydrophobic microporous membrane, operating at a low temperature, is utilized in membrane distillation systems to transfer water vapor solely from the feed side to the distillate side through membrane pores (Figure 4). As a result of gas-phase mass transfer, only volatile matter can pass through, resulting in nonvolatile matter being completely retained in the feed solution [70]. Membrane distillation bioreactors (MDBRs) integrate membrane distillation and conventional biological systems in a single reactor. The direct contact membrane module is submerged into the activated sludge tank. According to Wijekoon et al. [70], MDBRs have been studied for their ability to remove OMPs, and it was concluded that 95% of OMPs can be removed by this process, with biodegradation contributing to 70% of OMP removal. A detailed list of recently published MDBR studies is available in Pathak et al. [69].

## 3. Membrane Filtration for High-Quality Water Reuse

In today’s world, more than 2 billion people are facing severe water stress, while approximately 4 billion people experience severe water scarcity for at least one month each year. This pressing issue highlights the urgent need to explore alternative water sources, such as reclaimed water, especially in regions with inadequate water resource management practices [71]. The quality of reclaimed water plays a crucial role in water reuse, as it determines its suitability for specific purposes while ensuring safety and acceptability [72]. As emphasized by Foglia et al. [73], transforming conventional wastewater treatment plants (WWTPs) into reclaimed water facilities is necessary to promote safe water reuse practices and meet future water demands in water-scarce areas. To ensure human and environmental safety, it is essential to achieve superior water quality by removing toxic and persistent components. Therefore, extensive research on advanced membrane treatment technologies becomes imperative to achieve this objective. Hybrid dual-membrane systems, consisting of low-pressure membranes (MF/UF) followed by reverse osmosis (RO), have been proposed for treating wastewaters to obtain high-quality water for reuse purposes [74]. Moreover, innovative membrane treatment technologies that contribute to high-quality water reuse are also explored within this context.

### 3.1. Hybrid Dual-Membrane System

#### 3.1.1. MF/UF–RO Systems

**Figure 5:**
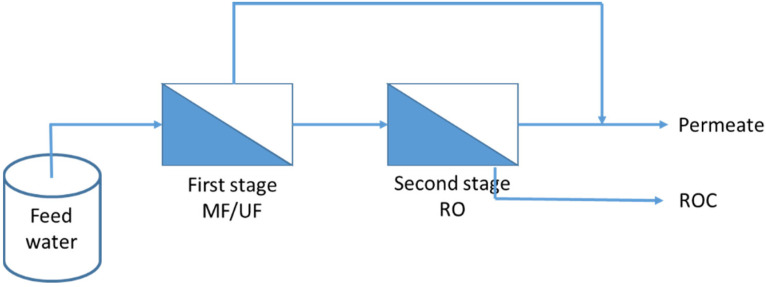
Schema showing a hybrid dual-membrane system for water reuse purposes.

In the field of municipal wastewater reclamation, the industry standard in many countries has shifted towards the adoption of dual-membrane systems, specifically combining MF/UF membranes followed by RO [74,75]. This approach, depicted in Figure 5, involves the initial treatment of feedwater using low-pressure membranes such as MF/UF, which effectively remove macromolecules, major foulants, and bacteria. Subsequently, the permeate obtained from the MF/UF process undergoes further filtration through RO, targeting the removal of micro-organics, mono- and divalent ions, metals, toxins, and other contaminants. This sequential membrane treatment ensures a comprehensive purification process for achieving high-quality reclaimed water.

Güneş and Gönder [76] conducted a study on a combined system of EC (electrocoagulation), NF (NF 270), and RO (BW 30) processes for treating biologically treated textile wastewater for reuse purposes. The EC pretreatment proved to be beneficial in terms of improving fluxes and reducing fouling. The NF 270 membrane exhibited excellent contaminant rejection, high fluxes, and low membrane fouling in the hybrid EC–NF process. The hybrid system, consisting of EC + NF 270 + BW 30, produced high-quality permeate suitable for reuse in all textile finishing processes.

Suwaileh et al. [77] explored the use of membrane distillation (MD) coupled with an electrodialysis (ED) system for producing high-quality water at a low cost, specifically for irrigation purposes. Additionally, Kim et al. [78] investigated a hybrid system combining fertilizer-drawn forward osmosis (FDFO) and nanofiltration (NF) to treat mine-impacted water. In terms of energy consumption, the FDFO–NF hybrid system demonstrated significantly lower energy requirements (1.08 kWh/m^3^) compared to MF–RO and UF–RO systems operating with similar feed solutions.

Šostar-Turk et al. [79] studied the treatment of wastewaters from reactive dye painting using combined UF and RO membranes in a pilot plant. They found that the UF membrane removed organic macromolecules from the wastewater; however, it failed to meet the discharge limits. The subsequent RO membrane was able to meet the discharge limits by further removing contaminants such as urea, sodium alginate, reactive dye, and oxidizing agents. Here, the UF membrane reduced the fouling effect of RO significantly by rejecting major foulants. Therefore, the application of membrane-based pretreatments such as MF and UF can significantly reduce fouling potential to a greater degree than conventional pretreatment processes [80].

The use of MF membranes prior to RO can significantly reduce the power costs of RO plants by up to 60%, as the membranes effectively manage the fouling of RO. Compared to traditional physicochemical treatments, an MF pretreatment can reduce turbidity by 60–80% and bring the silt density index (SDI) of the feed water below the minimum cut-off value of 3. In contrast, conventional pretreatment systems may only reduce the SDI index to 5–7 [81].

#### 3.1.2. Dual-Membrane Systems Used in the Real World

Numerous pilot wastewater treatment plants across the globe have explored the effectiveness of dual-membrane processes, including continuous microfiltration (CMF) followed by RO, for municipal wastewater reclamation and reuse. Examples of water reuse facilities that have implemented dual-membrane processes can be found in del Pino and Durham [82].

(a) Samsung Chemicals Co, Ltd., Daesan, Republic of Korea: Conventional pretreatment systems were unable to treat local polluted rivers below an SDI of 3. The subsequent RO post-treatment suffered membrane fouling. The installation of a Memcor CMF system prior to RO was able to improve the quality of RO feed, and treated 30,000 m^3^/d of polluted river water. The SDI of the effluent of the CMF system was less than 3, and RO operated more reliably [82].
(b) Vértesi Power Plant Co., Oroszlány, Hungary: The cooling lake situated next to the Vértesi Power Plant experienced a decline in water quality over the last decade. The lake’s total dissolved solids (TDSs), total suspended solids (TSSs), and algae content were reported to be 6000 mg/L, 100 mg/L, and 225 million counts/L, respectively, leading to an increase in deionizer chemicals and regeneration frequency; however, the implementation of CMF/RO prior to the deionizer effectively reduced the TDS level to 5–10 mg/L in the RO permeate, resulting in lower operation and maintenance costs as well as ion-exchange operation costs [82].
(c) The Tias WWTP, Lanzarote, Canary Islands, Spain: The WWTP utilized the USF Memcor CMF and RO systems to treat its effluent. The CMF system generated 1020 m^3^/d of filtrate that was devoid of suspended solids (<1.0 mg/L), turbidity (<1.0 NTU), and total and fecal coliforms. The SDI was <3.0, and the system achieved a water recovery rate of 85%. The 600 m^3^/d of microfiltered water was subsequently treated by FILMTEC BW30–400 RO membranes manufactured by Dow Chemicals (Midland, MI, USA), which generated 430 m^3^/d. The RO permeate of 600 m^3^/d (TDS content of 20 mg/L) and microfiltered water of 420 m^3^/d (TDS content of 1100 mg/L) were blended together and used for irrigation purposes [82].
(d) Water reclamation and management scheme (WRAMS) at Sydney Olympic Park, Australia: The WRAMS was designed to treat a mix of secondary effluent and stormwater. It consists of CMF and RO membrane filters, with a capacity of 7.5 ML/d. The permeate from the CMF and RO is mixed in an appropriate ration to produce reusable water and sold back to consumers [10,83].
(e) One of the largest wastewater treatment plants was established recently in Sulaibiya (Kuwait), where RO and UF-membrane-based membrane filtration is employed to reclaim municipal wastewater for nonpotable uses such as industry, irrigation, and aquifer recharge. The initial capacity of the plant was to produce treated water up to the volume of 375,000 m^3^ per day, and designed for future extension to 600,000 m^3^ per day [12].

To meet environmental standards effectively, the use of a dual-membrane system, consisting of CMF followed by RO, provides an efficient solution for the reclamation of water for nonpotable purposes. This system has excellent rejection capabilities, resulting in high-quality product water, but its main disadvantages are the high cost and energy consumption. Researchers are currently exploring alternative solutions to reduce energy consumption in such systems, with one area of in-depth investigation being the use of NF membranes instead of RO in indirect potable water reuse applications.

### 3.2. Innovative Membrane Treatment Technologies for High-Quality Water Reuse

In response to the escalating levels of water pollution that have surpassed the capabilities of existing RO membranes, there is an immediate requirement to develop advanced RO membranes with multidisciplinary features and superior performance to effectively remove salts and resist fouling [84]. Extensive efforts have been made to modify RO membranes, resulting in improved salt removal and enhanced resistance to fouling [22,23,24]. These advancements in RO membrane modification hold significant potential for various applications, particularly in small-scale water reclamation plants.

#### 3.2.1. NF as an Alternative to RO

According to recent research, alternative membrane filtration methods, such as NF and low-pressure RO (LPRO) membranes, appear viable substitutes for pressure-driven RO in integrated membrane systems. The use of these systems could reduce costs and energy consumption [85,86].

An economic analysis has shown that replacing RO membranes with NF membranes, specifically NF270 with a molecular cut-off of around 200 Da, could result in substantial annual cost savings ranging from USD 55,123 to USD 187,452 [85]. This would translate to a cost reduction of USD 0.07 per cubic meter of water treated, amounting to USD 53,000 per year for a 100 cubic meters per hour plant. Furthermore, the use of low-pressure and relatively low-fouling NF membranes such as NF-4040, which can sustain pressure three to four times less than that of a conventional RO membrane, can result in potential savings of USD 0.03 to USD 0.08 per cubic meter of treated reclaimed water [86].

Moreover, NF membranes, such as NF90 by Dow/Filmtec, exhibit similar trace organic rejection rates as RO membranes [87] and meet up to 96% of overall drinking water standards, with the exception of boron, molybdenum, and ammonia [88].

A comparative analysis of low-pressure RO (LPRO) and NF membranes demonstrated that LPRO/NF membranes can eliminate over 80% of TOC and 60–100% of conductivity [85]. With regard to micropollutant removal, the LPRO permeate contained two micropollutants (atenolol and TCEP), whereas the NF270 permeate contained ten out of the seventeen micropollutants present in the feed water. Most of the negatively charged pollutants, such as diclofenac, gemfibrozil, and ketoprofen, were not found in the NF270 permeate [85]. This observation was explained by Verliefde et al. [89], who concluded that negatively charged NF/RO membranes are less effective at removing positively charged pollutants (atenolol and TCEP) due to electrostatic repulsion.

Issues of NF: Fouling and scaling and the incomplete removal of contaminants are seen as issues of NF in some instances. NF/RO membranes are susceptible to fouling by various particulate contaminants present in the feedwater, including refractory organics, trace amounts of synthetic organics generated during disinfection processes, and soluble microbial products derived from biological treatments [90]. The removal of charged pharmaceuticals, such as diclofenac and salicylic acid, was effectively accomplished by both NF (92%, 93%) and RO membranes (92%, 95%). In contrast, noncharged compounds, such as 2-Naphtol, Bisphenol-A, Phenacetine, and Primidone, were less effectively rejected by NF (12%, 45%, 19%, 87%) and RO (43%, 99%, 71%, 84%) membranes [91].

#### 3.2.2. Membrane Hybrid Systems as Pretreatments to NF

An NF membrane alone in the reclamation of sewage is limited by membrane fouling due to organics. Meier and Melin [92] investigated the coupling of PAC prior to NF (PAC–NF) in the treatment of sewage, and reported that the adsorbent layer deposited on the membrane surface reduced NF permeability. They further suggested using UF–PAC or MF–PAC prior to NF to improve membrane permeability. 

In a previous study [93] a PAC–NF hybrid system was employed to treat biologically pretreated landfill leachate. In this system, PAC was added to the feed of the NF unit and had a positive impact on the quality of permeate, permeate flux, and fouling layer in the NF membrane. Although the PAC–NF system required less operating pressure and energy consumption compared to RO, the concentration of contaminants in the permeate was higher than that of the RO permeate, although still within the acceptable effluent quality standards.

**Figure 6:**
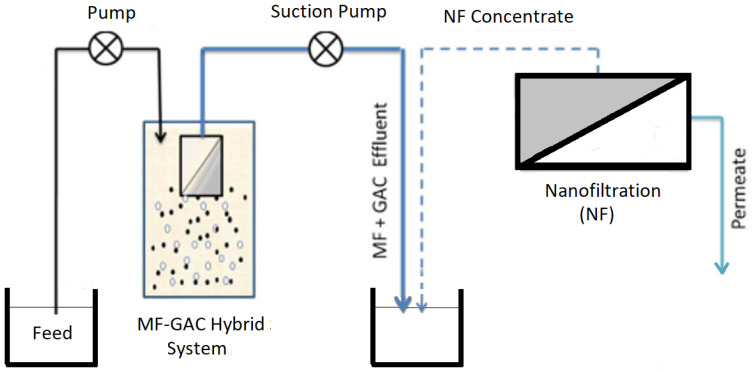
Schematic diagram of MF–GAC hybrid system followed by NF (modified and redrawn from [94]).

A study conducted later [94] investigated the performance of a combined MF–GAC hybrid system followed by NF in removing organics (Figure 6). The MF–GAC (with an initial GAC dose of 2 g/L and GAC daily replacement of 10%) system removed 53% of organics, whilst the combination of the MF–GAC system and NF achieved more than 95% rejection [94]. This eventually extended the NF membrane lifespan and improved NF membrane permeability. 

In the presence of hardness in the feed water, NF is used as a pretreatment step to lower the levels of Ca and Mg ions. This helps to reduce the scaling effect on the RO membrane [95], particularly for low-quality seawater and brackish water that require more extensive pretreatment prior to RO [25].

#### 3.2.3. NF as Pretreatment to RO

NF has been found to effectively remove Ca and Mg ions, thereby reducing scaling on RO membranes [96]. Llenas et al. [97] conducted a study on the feasibility of using NF before RO in seawater desalination to prevent scaling effects on RO membranes. They found that most of the six NF membranes tested rejected 90% of sulfate ions, resulting in a significant reduction in scaling effects. Additionally, the use of NF as a pretreatment step prior to RO maximized the reduction in PPCPs.

**Figure 7:**
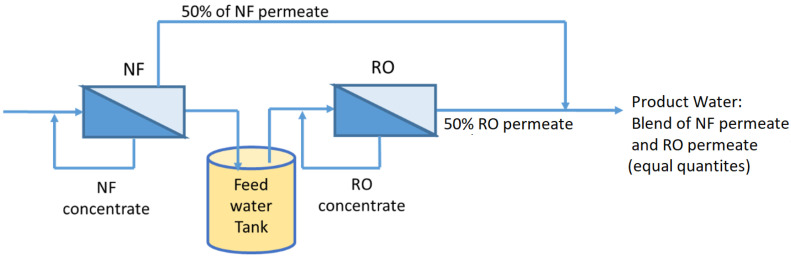
NF as pretreatment to RO for high-quality water reuse (modified and redrawn from Shanmuganathan et al. [95]).

Shanmuganathan et al. [95] investigated whether BTSE could be treated using nanofiltration (NF) and reverse osmosis (RO) to reduce the SAR (sodium absorption ratio) to within the safety limits for irrigation purposes. It was reported that an NF–RO hybrid system can produce water suitable for irrigation purposes, especially for sensitive crops. The product water can be obtained in such a way by mixing 50% of an NF permeate and 50% of an NF–RO permeate together to produce the required SAR (Figure 7). Furthermore, as per a membrane autopsy, the use of NF prior to RO reduced the organic deposition on the RO; therefore, the lifespan of RO can be extended. 

In a study by Hassan et al. [98], the integration of NF with SWRO and MSF resulted in the removal of over 90% of divalent ions and approximately 40% of monovalent ions. The high quality of water produced by NF led to a significant increase in water recovery, with SWRO and MSF reaching 70% and 80%, respectively, and a reduction in energy consumption by 25–30% [99]. Consequently, the integration of NF–SWRO led to a significant improvement in seawater desalination processes, with the SWRO recovery ratio doubling.

#### 3.2.4. Track-Etched Membranes (TeMs) in Membrane Distillation

Yeszhanov et al. [100] conducted a review on a new type of membranes, known as track-etched membranes (TeMs), which have been utilized for water treatment to remove salts, pesticides, and traces of liquid radioactive waste in membrane distillation. TeMs have a narrow pore size, precise number of pores per unit area, and a narrow thickness with a tortuosity of 1. These characteristics allow for accurate and efficient water purification, particularly in the removal of low-level liquid radioactive waste. TeMs are also used as model membranes with which to test theoretical models.

In a subsequent publication by the same authors [101], modified PET track-etched membranes were introduced. These membranes were hydrophobized via the photoinduced graft polymerization of stearyl methacrylate inside the pores, resulting in enhanced emulsion separation with stable fluxes. The membranes with larger pore sizes exhibited a maximum flux of 1100 mL/m^2^∙s for chloroform–water emulsion at a vacuum pressure of 900 mbar. A hydrophobic membrane with a pore size of 3.05 μm (pore density of 1 × 10^6^) exhibited a three-fold increase in flux compared to a membrane with a pore size of 350 nm (pore density of 1 × 10^8^).

## 4. Recent Membrane Technologies for ROC Management

Previous studies that have reported on the treatment of ROCs using membrane-based treatment systems to remove refractory organics are summarized below.

### 4.1. NF Membranes

Mousavi and Kargari [102] conducted a study on the use of three different types of NF membranes (TW30, NE90, and NE70) for treating the RO concentrate generated by a petrochemical complex. The TW30 membrane showed excellent performance in removing total dissolved solids (TDSs), with a 93% rejection rate at a flux of 2.84 L·m^−2^·h^−1^·bar^−1^ at 10 bar. The TW30 membrane also demonstrated high removal rates for divalent ions such as calcium (96.1%) and magnesium (98.7%), while chloride removal was 90.3%. The NE90 and NE70 membranes showed lower removal rates, but exhibited higher flux rates.

### 4.2. RO–NF Treatment System

**Figure 8:**
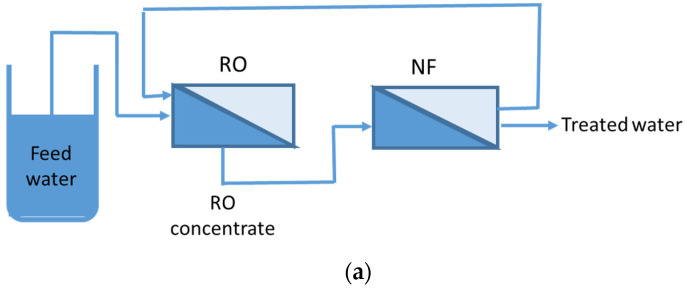

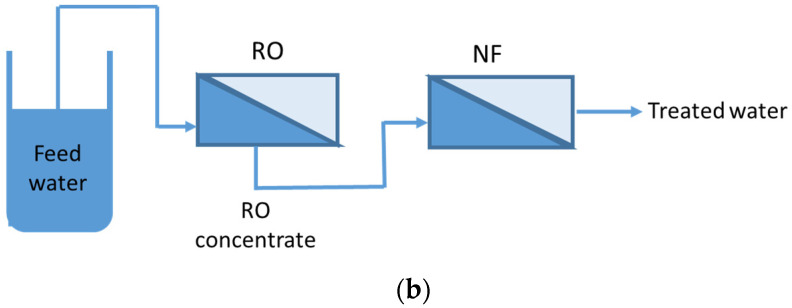
Experimental set-up of (**a**) RO–NF, and (**b**) NF-RO hybrid system (modified and redrawn from Afrasiabi and Shahbazali [103]).

Afrasiabi and Shahbazali [103] demonstrated the use of an RO–NF hybrid system for the treatment of ROCs (Figure 8). This hybrid configuration improved water recovery, increasing it from 37% to 85%. The RO–NF coupling also reduced energy consumption and increased the quantity of produced water.

Ali [104] found that the NF treatment applied for the removal of divalent ions from the brine achieved salt rejection in the order of CaSO_4_ (97.4%), Na_2_SO_4_ (97.3%), MgSO_4_ (95.2%), MgCl_2_ (93.4%), and NaCl (79%). This minimized the load on additional stages of RO membranes.

### 4.3. MF–GAC Hybrid System/NF–RO

Shanmuganathan et al. [48,105] conducted short-term and long-term experiments with an MF–GAC hybrid system, with varying doses of GAC, to investigate the removal of organics from ROCs sourced from a water reclamation plant in Sydney, Australia. The MF–GAC hybrid system with 5 g/L GAC was able to remove 20–60% of DOC. The removal of hydrophobics and hydrophilic was 42% and 46%, respectively, after 6 h of operation. An increase in GAC to 20 g/L led to the removal of 85% of DOC [48].

An MF–GAC hybrid system was operated for an experiment conducted for 10 days to investigate the removal of organics and trace organics from RO concentrated [105]. The initial GAC dose was 10 g/L, with 10% daily GAC replacement. The DOC reduced by 60–80% over the operational time of 10 days. There was an organic micropollutant removal rate of 89–99% by the end of the experiment. 

**Figure 9:**
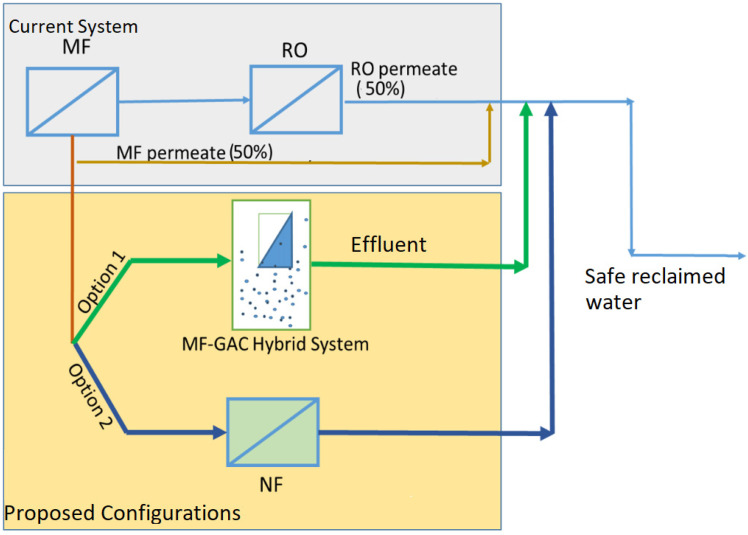
Membrane system for the WTP to produce safe reclaimed water (modified and redrawn from [10]).

In order to effectively eliminate the different types of micropollutants present in ROCs, it is crucial to establish sustainable hybrid systems. According to Devaisy et al. [10], two effective methods for removing micropollutants from ROCs are an MF–GAC hybrid system or NF treatment. Both methods were found to be equally effective in removing these compounds. The authors recommend a design that incorporates the permeate from either an MF–GAC hybrid system or NF membrane (NTR 729HF) filtration with RO permeate. This design is illustrated in Figure 9.

#### Cost Comparison: NF–RO Combination Versus Two-Stage RO

Incorporating NF as a pretreatment step before RO offers several advantages: Firstly, it reduces the pressure requirements for RO, leading to energy savings. Additionally, NF is effective in removing a significant portion of low-molecular-weight organics, thereby minimizing organic and biofouling during the subsequent RO process. This reduction in fouling helps to extend the lifespan of RO membranes, reducing the need for frequent membrane replacements and lowering overall energy costs [106].

Another promising approach is the utilization of a submerged membrane adsorption hybrid system, which successfully removes effluent organic matter and emerging pollutants from wastewater. This system not only improves the quantity of recycled water available for irrigation reuse but also enriches it with valuable nutrients. An economic analysis of the membrane GAC adsorption hybrid system has demonstrated its cost-effectiveness for organic and micropollutant removal [106]. The quantity of GAC required for treating biologically treated sewage effluent is relatively small (less than 50–100 g/m^3^ of water produced), and the main operational cost lies in periodic GAC replacement. The cost of GAC treatment for 1 m^3^ of sewage effluent is approximately USD 0.05 per m^3^. Considering the adverse environmental impacts of discharging untreated wastewater into receiving waters, the cost of USD 0.05 per m^3^ of wastewater treated is reasonable [5]. Furthermore, employing the submerged membrane adsorption hybrid system as a pretreatment step helps to mitigate biofouling during the subsequent RO process, leading to energy savings in RO operations.

### 4.4. Forward Osmosis with GAC Pretreatment

Jamil et al. [107] investigated the use of forward osmosis (FO) to minimize the volume of ROCs and showed that a volume of 6–8 L of ROCs was reduced by 8% in five repeated FO steps using a draw solution (DS) of 2 or 3 M NaCl; however, they also observed a decrease in flux due to membrane fouling and scaling as the concentration of ions increased in the DS. To further enhance micropollutant removal, the authors added granular activated carbon (GAC) pretreatment for fixed-bed adsorption to the FO system.

### 4.5. Membrane Distillation (MD) in Wastewater Reverse Osmosis Concentrate (WWROC) Treatment

Researchers have investigated the use of membrane distillation (MD) for treating ROC discharged from wastewater reclamation plants (WRPs) [108]. In a detailed experiment using direct contact MD (DCMD) with ROCs, researchers achieved 85% recyclable water recovery with only a 13–15% decline in flux and good-quality permeate (10–15 µS/cm, 99% ion rejection) at a moderate feed temperature of 55 °C. The low salinity and loose CaCO_3_ adhesion on the membrane did not significantly contribute to DCMD flux decline; however, the organic content in ROCs (58–60 mg/L) resulted in a significant reduction in membrane hydrophobicity (70% lower water contact angle than a virgin membrane). To address this issue, researchers used granular activated carbon (GAC) pretreatment to reduce the organic content of ROCs by 46–50%. GAC pretreatment effectively adsorbed a majority of organic matter, including micropollutants, resulting in high-quality water production by MD and improved reuse potential. The MD concentrated ROCs were also suitable for selective ion precipitation, which could lead to near-zero liquid discharge in water reclamation plants.

### 4.6. Direct Contact Membrane Distillation (DCMD) and Freeze Crystallizer (FC)

The impact of direct contact membrane distillation (DCMD) and freeze crystallizer (FC) on treating ROCs was investigated by Naidu et al. [109]. The study found that DCMD achieved a water recovery rate of 60% with pretreated ROCs. The use of chemical pretreatment enhanced the removal of Ca ions by more than 95%, resulting in a significant improvement in flux in cycles one and two. On the other hand, FC achieved a water recovery rate of 56–57% in multistage freezing with freshwater ice (TDS < 80–370 mg/L).

### 4.7. Selectrodialysis with Bipolar Membranes (BMSED)

According to Chen et al. [110], a novel process called selectrodialysis with bipolar membranes (BMSED) was utilized to treat RO concentrate. This process combines selectrodialysis (SED) and bipolar membrane electrodialysis (BMED), which employs bipolar membranes and ion-exchange membranes (monovalent-selective) inside the ED stack in a single step. This system can remove monovalent ions from RO concentrate, and selectively regenerate monovalent ions as well as produce acids (HCl) or bases (NaOH) in a single step.

In long-term operation, it was shown that 105 g/L of RO concentrate was desalinated and converted into NaOH or HCl byproducts, and their respective concentrations were increased to 2.2 and 1.9 mol/L. The purities of these byproducts were nearly 99.99%. The BMSED not only treats the RO concentrate but can also reclaim it as an acid or alkaline byproduct, which can be used for industrial purposes.

Different types of membrane treatment systems used to treat ROCs are given in Table 2.

## 5. Conclusions

Water scarcity is a pressing global issue, and the reuse of wastewater offers a promising solution to meet the increasing demand for water. Membrane technology plays a crucial role in producing high-quality water suitable for both domestic and industrial reuse applications. Integrating physicochemical treatments with membranes has proven effective in removing contaminants while minimizing energy consumption.

Extensive research has been conducted on various aspects of membrane treatment, including pretreatment techniques, hybrid membrane systems, hybrid dual-membrane systems, and innovative membrane technologies. These studies have aimed to enhance contaminant removal and improve water recovery efficiency. Additionally, membrane-based treatments have been explored for managing membrane-rejected concentrates (ROCs), focusing on improving contaminant removal and maximizing water recovery.

To facilitate the widespread adoption of high-quality water reuse, particularly in developing countries with limited resources, it is crucial to conduct further research and development. This review emphasizes the importance of such research endeavors to benefit communities in third-world countries, where affordable solutions for high-quality water reuse are of the utmost importance. By addressing the specific challenges faced by these communities, advancements in membrane technology can contribute significantly to sustainable water management and improve quality of life. The reuse of wastewater presents one of the best options to meet the growing demand for water worldwide. Membrane technology plays a vital role in producing high-quality water for both domestic and industrial reuse.

## Figures and Tables

**Table 1:** Pretreatments applied prior to membrane filtration to enhance membrane performance.

No.	Pretreatment and Experimental System	Details of Membranes	Results		Refs.
			Without Pretreatment	With Pretreatment	
1.	Effect of coagulation on the performance of MF and UF; Coagulant was Fe. Experimental system: coagulation followed by MF; coagulation followed by UF. Feed water: artificial seawater which has NTU = 10 and pH = 8.	MF: hollow fiber; polyvynilidenefluoride (PVDF) material; pore size of 0.1 μm; and initial flux (L/m^2^ h at 50 kPa) of 1100	Silt density index (SDI_15_) After MF = 3.17	SDI_15_ after coagulation followed by MF = 0.75	[29]
		UF: hollow fiber; PVDF material; pore size of 0.05 μm; and initial flux (L/m^2^ h at 50 kPa) of 350	Silt density index (SDI_15_) After UF = 2.76	SDI_15_ after coagulation followed by UF = 1.88	
2.	Effect of coagulation on the performance of MF; *Moringa oleifera* (MO) and alum were used as coagulants. Feed water: river water; Turbidity: 7.8 NTU; Color: 8.7 PCU.	MF: hollow fiber; polyvynilidenefluoride (PVDF) material; pore size of 0.1 μm; and permeate flux (140 L/m^2^ h)	MF without coagulation; TMP development from 12.0 to 27 kPa over 3 h	MO (2 mL/L)—MF: TMP development from 12.0–16.5 kPa over 6 h Alum-MF: TMP development from 12–14.5 kPa over 6 h.	[33]
			Removals of contaminants by MF only: Turbidity (NTU) = 0.2 ± 0.1; Color (PCU) = 1.9 ± 0.1.	Removals by MO-MF treated: Turbidity (NTU) = 0.0; Color (PCU) = 0.3 ± 0.1. Removals by Alum-MF treated: Turbidity (NTU) = 0.0; Color (PCU) = 0.0 ± 0.0.	
3.	The effect of coagulations such as alum (Al_2_(SO_4_)_3_) on the membrane permeability. Feed water: wastewater discharged from Wood processing facility	UP150 Microdyn Nadir™ Polyethersulfone (PES) Hydrophilic membrane at 10 bar; MWCO (da) ∼150,000; Water flux < 570 LMH/2 bar.	At 3 bar, the flux declined from 30 LMH to 5 LMH over 120 min.	Improved flux after treated by alum: 110 LMH—25 LMH over 120 min.	[36]
		NF270 DOW Filmtec Polyamide (PA) Hydrophilic 41 bar MWCO (Da) ∼200−400 Water flux 122−167 LMH/8.8 bar.	At 15 bar, the flux declined from 50 LMH to 10 LMH over 120 min.	Improved flux after treatment by alum: 80 LMH to 20 LMH over 120 min.	
		NF90 membrane DOW Filmtec Polyamide (PA) Hydrophilic 41 bar MWCO (Da) ∼200−400 Water flux 78−102 LMH/8.8 bar.	Fouling rate was 80% with NF only.	Fouling rate was 55% after treatment with alum; 55% after treated with *Moringa Oleifera* powder.	
4.	The effect of oxidation, ozonation, and ion exchange followed by UF; Feed water: lake water.	UF membrane: polyvinylidene fluoride in a stirred cell of dead-end configuration; 0.1 μm pore size; and hydrophobic nature.	UF only: Unified membrane fouling index (UMFI) (m^2^/L) of 0.22.	Pretreatments such as UV/H_2_O_2_, ozonation, and AER reduced UMFI (m^2^/L) to 61%, 43%, and 23%, respectively.	[30]
5.	The effect of an ion-exchange resin followed by MF in a hybrid system; Feed water: RO concentrate.	Hydrophilic modified Polyacrylonitrile (PAN), nominal pore size of 0.10 μm; surface are of 0.2 m^2^; manufactured by MANN + HUMMEL ULTRA-FLO PTE LTD, Singapore.	MF only: TMP development from 100 mbar to 350 mbar over 400 min of operation. Removal of DOC < 10%.	Pretreatment reduced the TMP development from 100–250 mbar over 400 min; DOC removal 55–63% at the ion-exchange resin (Purolite^®^ A502PS).	[45]
6.	The effect of advanced oxidation (ozonation) and biologically activated carbon (BAC) on the subsequent RO permeability. Feed water: secondary effluent of a wastewater treatment plant.	A ceramic MF membrane (Pall^®^, 0.1 μm, ZrO_2_); Filmtec^®^ BW30 membranes; BAC column (the activated carbon, Acticarb BAC GA1000N).	RO normalized flux Without pretreatment = 0.79;	RO normalized flux After ozonation + MF = 0.80; After BAC = 0.82; After ozonation + MF + BAC = 0.84.	[31]

**Table 2:** Treatment of ROCs using membrane-based treatments.

	Experimental System	Membranes	Performance of the System in Terms of Removal Efficiency	Refs.
1.	NF membranes Feed: petrochemical complex	TW30	TDS = 93% Divalents = 96–98.7% Chloride ions = 90.3%	[102]
2.	MF–GAC hybrid system Feed: wastewater treatment	NTR 729HF (polyvinylalcohol/polyamides, 700 Da) RO membrane (polyamides) by Woongjin Chemical (100 Da)	DOC = 60–80% Micropollutants = 89–99%	[10]
	NF–RO hybrid system Feed: WWTP		Similar removals as above	
3.	FO to minimize the volume of ROCs Feed: WWTP	FO membrane: cellulose triacetate material with embedded polyester screen support (CTA-ES membrane 1401270); pore size = 0.74 nm	Volume reduction = 8% in five repeated steps Draw solution: 2–3 M NaCl	[107]
4.	Membrane distillation with GAC pretreatment Feed: WWTP	A hydrophobic polytetrafluoroethylene (PTFE) flat sheet membrane (General Electric, Boston, MA, USA); pore size = 0.2 µm	Water recovery (recyclable) = 85%; good permeate quality (EC = 10–15 µS/cm; ion rejection 99%) Flux decline of Raw ROCs = 20–25% Pretreated ROCs (13–16%)	[108]
5.	Direct contact membrane distillation and freeze crystallizer (DCMD and FC) Feed: seawater desalination plants	A commercial hydrophobic polytetrafluoroethylene (PTFE) flat sheet membrane; pore size = 0.2 μm	Water recovery of DCMD = 60%; chemically pretreated ROCs enhanced the performance of DCMD; and water recovery of FC in a multistage freeze/thaw approach = 56–57% and good-quality freshwater ice with TDS < 0.08–0.37 g/L	[109]
6.	Selectrodialysis with bipolar membranes (BMSED): Feed: seawater desalination plants	ASTOM ASV/CSO and Selemion ACS/CIMS monovalent selective membranes	Permaselectivity of Na^+^/Ca^2+^ and Cl^−^/SO_4_^2−^ ranged from 5–10 and 50–60 Formation of NaOH and HCl byproducts with a purity of 99.99%	[110]

## Data Availability

Not applicable.
